# Effects of Acute Prenatal Exposure to Ethanol on microRNA Expression are Ameliorated by Social Enrichment

**DOI:** 10.3389/fped.2014.00103

**Published:** 2014-09-24

**Authors:** Cherry Ignacio, Sandra M. Mooney, Frank A. Middleton

**Affiliations:** ^1^Department of Neuroscience and Physiology, State University of New York Upstate Medical University, Syracuse, NY, USA; ^2^Department of Biochemistry and Molecular Biology, State University of New York Upstate Medical University, Syracuse, NY, USA; ^3^Developmental Exposure Alcohol Research Center (DEARC), Binghamton University, Binghamton, NY, USA; ^4^Department of Pediatrics, University of Maryland School of Medicine, Baltimore, MD, USA

**Keywords:** fetal alcohol syndrome, social behavior, amygdala, ventral striatum, adolescence, gene expression, next generation sequencing

## Abstract

Fetal alcohol spectrum disorders (FASDs) are associated with abnormal social behavior. These behavioral changes may resemble those seen in autism. Rats acutely exposed to ethanol on gestational day 12 show decreased social motivation at postnatal day 42. We previously showed that housing these ethanol-exposed rats with non-exposed controls normalized this deficit. The amygdala is critical for social behavior and regulates it, in part, through connections with the basal ganglia, particularly the ventral striatum. MicroRNAs (miRNAs) are short, hairpin-derived RNAs that repress mRNA expression. Many brain disorders, including FASD, show dysregulation of miRNAs. In this study, we tested if miRNA and mRNA networks are altered in the amygdala and ventral striatum as a consequence of prenatal ethanol exposure and show any evidence of reversal as a result of social enrichment. RNA samples from two different brain regions in 72 male and female adolescent rats were analyzed by RNA-Seq and microarray analysis. Several miRNAs showed significant changes due to prenatal ethanol exposure and/or social enrichment in one or both brain regions. The top predicted gene targets of these miRNAs were mapped and subjected to pathway enrichment analysis. Several miRNA changes caused by ethanol were reversed by social enrichment, including mir-204, mir-299a, miR-384-5p, miR-222-3p, miR-301b-3p, and mir-6239. Moreover, enriched gene networks incorporating the targets of these miRNAs also showed reversal. We also extended our previously published mRNA expression analysis by directly examining all annotated brain-related canonical pathways. The additional pathways that were most strongly affected at the mRNA level included p53, CREB, glutamate, and GABA signaling. Together, our data suggest a number of novel epigenetic mechanisms for social enrichment to reverse the effects of ethanol exposure through widespread influences on gene expression.

## Introduction

Prenatal ethanol exposure can cause fetal alcohol spectrum disorders (FASDs). With 30% of all women reporting drinking alcohol at some time during pregnancy (1), FASD prevalence in the US and some Western European countries is estimated at 2–5% of school children (2). FASDs are associated with impaired learning and memory, language development, and abnormal social behavior [reviewed in Ref. (3)]. The social behavior changes seen in adolescents can resemble those that are typically associated with autism.

Behavioral deficits can also be seen in animal models of prenatal ethanol exposure. Acute exposure on gestational day 12 (G12) in rats leads to decreased social investigation and play fighting, as well decreased social motivation in late adolescence and adulthood (4, 5). To date, amelioration of social behavior deficits from prenatal ethanol exposure has largely focused on behavioral interventions. However, social experience with typically developing peers has been found to be important for improving social skills and increasing social interaction in autistic children. In previous work, we showed that a form of social enrichment (housing ethanol-exposed rats with non-exposed control rats) could normalize the social motivation deficit phenotype seen in both males and females at postnatal day 42 (P42) following gestational ethanol exposure at G12 (5).

The amygdala is critical for normal social behavior. Lesions of the amygdala alter social functions in human beings and experimental animals (6), and developmental changes in the amygdala have been described in autism (7). The amygdala is thought to regulate social behavior in part through connections with the prefrontal cortex, thalamus, and basal ganglia (8, 9). Within the basal ganglia, the ventral striatum has been viewed as a critical integration center for social/emotional signals from the amygdala, as well as spatial/contextual information from the hippocampus, reward/motivational signals from midbrain dopamine neurons, and cognitive signals from the prefrontal cortex (9).

At the cellular level, the amygdala is composed of a group of 13 sub-nuclei located in the medial temporal lobe (8). These nuclei may be divided into four subdivisions (10): (1) basolateral (which includes the lateral, basolateral, and basomedial nuclei), (2) cortical like (including nucleus of the lateral olfactory tract, bed nucleus of the accessory olfactory tract, the cortical nucleus, and the periamygdaloid cortex), (3) centromedial (central and medial nuclei, and the amygdaloid part of the bed nucleus of stria terminalis), and (4) other (which includes anterior amygdala area, the amygdalo–hippocampal area, and the intercalated nuclei). Developmentally, many amygdala nuclei derive from the medial ganglionic eminence (i.e., are diencephalic) (11), although the cortical amygdaloid nuclei are telencephalic in origin (12). Neuronal types differ considerably among the subdivisions of the amygdala (10). In the basolateral group, approximately 70% of neurons are thought to be glutamatergic (pyramidal, spiny, or class I neurons). This division also contains interneurons such as GABAergic non-spiny stellate cells of the cortex (called S cells, stellate, or class II neurons). In contrast, within the central nucleus, the majority of cells are thought to be GABAergic.

microRNAs (miRNAs) are a class of short, hairpin-derived RNAs that repress gene expression at the post-transcriptional level. Mature miRNAs of ~20 nt in length canonically bind to complementary sequences found in the 3′ untranslated region of messenger RNAs (mRNAs), thereby repressing translation by ribosomes. In neurons, miRNAs also play a role in compartmentalizing specific mRNA translation in subcellular components, including axons (13) and synapses [reviewed in Ref. (14)]. Dysregulation of miRNAs have recently been associated with a variety of neurodegenerative diseases as well as alcohol consumption in human beings (15) and rodent fetal exposure models [reviewed in Ref. (16)].

In this study, we extend our previous characterization (5) of selected alterations in gene expression in the amygdala and ventral striatum as a consequence of prenatal ethanol exposure and an environmental manipulation (social enrichment) in rats. Using the same tissue samples used in our previous study (5), we analyzed miRNA from the amygdala and ventral striatum of 72 adolescent male and female rats. Samples were pooled to 24 for each brain region and analyzed by RNA-Seq and Affymetrix miRNA arrays. We identified many miRNAs with nominally significant changes due to prenatal ethanol exposure or social enrichment. Some of the gene expression changes due to ethanol were reversed by social enrichment. Pathway enrichment analysis was also performed on the top changed miRNAs. We comprehensively integrate these findings with our existing mRNA data to determine whether the target mRNAs of the altered miRNAs showed evidence of changing, using whole transcriptome microarray data from the same rats. Further, we broaden our mRNA analysis by considering all possible genes in the context of canonical pathways related to brain function. This additional analysis highlights striking reversals following social enrichment in p53, CREB, glutamate, and GABA signaling. Altogether, these analyses suggest possible mechanisms for social enrichment to reverse some of the effects of prenatal ethanol exposure.

## Materials and Methods

### Animals

Treatment of animals, as well as behavioral and mRNA expression outcomes, were described in Middleton et al. (5). Briefly, timed pregnant Long Evans rats (Harlan, Indianapolis, IN, USA) were received on G4, with G1 designated as the first day on which a sperm-positive plug was noted. These rats were housed at the Department of Veterans Affairs Medical Center (VAMC) in a facility accredited by the Association for Assessment and Accreditation of Laboratory Animal Care (AAALAC) in Syracuse, NY. All procedures were approved by the Institutional Animal Care and Use Committees at both the Syracuse VAMC and SUNY Upstate Medical University, and were in accordance with the guidelines for animal care established by the National Institutes of Health. Rooms were maintained on a reverse 12-h light/dark cycle at 22°C (lights off at 7:00 a.m.).

**Figure 1:**
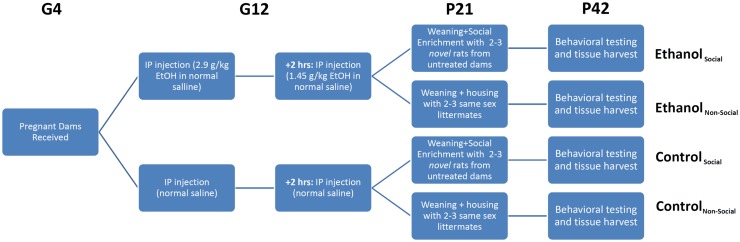
**Experimental overview**. Timed pregnant female rats were received on gestational day 4 (G4) and split into two treatment groups – ethanol (EtOH) or saline. Each animal was injected twice on G12 with EtOH or an equivalent volume of saline. Pups were delivered in their home cage, litters culled to 10 pups with a 1:1 ratio of males:females, and remained in their home cage until weaning. On weaning, rats were separated into non-social and social-enriched conditions for 3 weeks (P21-42), during which they were housed with 2–3 age- and sex-matched rats from their own litter or a novel litter from an untreated dam, respectively. All animals underwent behavioral testing using the social interaction test (SIT), which measures social investigation, contact behavior, play fighting, and social motivation (social preference vs avoidance for the novel rat). After testing, rats were euthanized and tissue harvested for RNA profiling.

Animals were exposed to ethanol prenatally as described previously (5). On G12, dams received an initial intraperitoneal (i.p.) injection of ethanol (2.9 g/kg as a 20% v/v solution in physiological saline) followed by a second i.p. injection 2 h later of 1.45 g/kg ethanol (Figure 1). Control animals received i.p. injections equivalent volumes of saline at the same timepoints. This method of ethanol administration leads to blood ethanol concentrations of 287 ± 3.5 mg/dl within 15 min of the second injection. After birth, all litters were culled to 10 pups within 24 h, with equal ratios of males/females as best as possible. On P21, litters were weaned and male and female offspring were housed separately. After social behavior testing (described below), animals were injected intraperitoneally with 100 mg/kg ketamine and 10 mg/kg xylazine prior to decapitation. Brains were rapidly removed, snap-frozen on dry ice, and stored at –80°C until used for RNA extraction (see below).

Our previous behavioral study on the same cohort of rats, described in Middleton et al. (5), examined the effects of a form of environmental manipulation termed social enrichment, during the post-weanling and early adolescent period (P21–P42) in animals prenatally exposed to ethanol (Figure 1). This treatment involved housing experimental animals (offspring of saline- or ethanol-injected dams) with either 2 or 3 same-sex littermates (non-enriched condition) or 2 or 3 novel same-age, same-sex animals from a non-treated dam (social enrichment condition). The effect of this manipulation on social behavior was evaluated on P42 by testing their responses to the introduction of another same-age, same-sex rat (from an untreated dam) over the course of a 10-min social interaction test (SIT). Among the behavioral measures assessed during the SIT were social investigation (sniffing of the novel rat’s body), contact behavior (grooming, crawling over or under the novel rat), play fighting (following, chasing, nape attacks, pinning), and social motivation (a coefficient of social preference vs avoidance of the novel rat). The most significant finding from our prior analysis was the finding that prenatal ethanol exposure negatively affected social motivation performance in both male and female rats following prenatal ethanol exposure, but this impairment was completely reversed by social enrichment.

### Molecular profiling

The present study was designed to determine the potential molecular substrates of decreased social motivation following prenatal ethanol exposure and its reversal by social enrichment. We first dissected the whole amygdala and ventral striatum from a total of 72 42-day-old male and female rats, using established anatomical landmarks, as described previously (5). After isolating the regions of interest, total RNA was purified using the RNeasy kit (Qiagen, Valencia, CA, USA). RNA yield and quality were assessed by UV spectrophotometry and the Agilent Technologies Bioanalyzer. A total of 144 RNA samples were purified from the 72 rats, including 9 males and 9 females for each of the 4 treatment groups: (1) ethanol-exposed/non-enriched, (2) ethanol-exposed/socially enriched, (3) saline-exposed/non-enriched, and (4) saline-exposed/socially enriched. For all subsequent molecular assays described in this report, three pools of RNA were created for each brain region using equal amounts of RNA from the nine male or nine female rats within each treatment group. Thus, a total of 24 RNA samples from each brain region were examined (12 male pools, 12 female pools), representing a total of 6 per treatment condition. We point out that this pooling strategy preserved our ability to look at the contribution of different brain regions and genders on expression level, and was made purely to reduce cost.

High-resolution quantification of miRNA expression was performed using small RNA-sequencing from 1 μg of each pool of total RNA according to the TruSeq Small RNA Sample Prep kit (Illumina, San Diego, CA, USA). Subsequent purification methods including gel purification of small RNAs 20–30 nt in length, resulted in more than 90% of all reads in the sample attributed to miRNAs. Libraries were indexed and multiplexed in sets of 8 (6 sets total) prior to sequencing (single-end, 37 cycles) using Reagent Kit v3 reagents on a MiSeq Benchtop Sequencer (Illumina, San Diego, CA, USA). Raw sequence FASTQ files were imported into Partek Flow software for initial analysis. Base calls below a phred score of 20 were trimmed from the reads. These were then aligned to the Rn5 version of the rat genome using the Bowtie algorithm (17). The aligned reads were then quantified against the miRBase 21 transcript annotations for both precursor and mature miRNAs (18). Reads from miRNA genes were normalized and scaled to reads per million (RPM) for comparison between samples and comparison with the microarray data in Partek Genomics Suite.

In order to validate the changes seen by RNA-Seq, we also examined the samples using the GeneChip miRNA 2.0 array (Affymetrix, Santa Clara, CA, USA). Samples from the same pooled RNA were prepared using the FlashTag Biotin HSR RNA Labeling Kit (Affymetrix, Santa Clara, CA, USA). Arrays were hybridized, washed, stained, and scanned according to manufacturer protocol and the data exported and normalized using RMA in Partek Genomics Suite.

We also examined the relationship of the miRNA data to our previously described mRNA dataset generated from the same pooled rat brain RNA samples; see Ref. (5). Those data were generated with the Rat ST Gene 1.0 array (Affymetrix, Santa Clara, CA, USA), normalized using RMA and imported into Partek Genomics Suite for analysis alongside the miRNA microarray and RNA-Seq data. We point out that while our previous report focused only on a subset of 663 mRNAs related to 17 gene ontology terms of interest (social, anxiety, fear, autism, synapse, synaptic, norepinephrine, neuropeptide, cannabinoid, opioid, oxytocin, GABA, glutamate, glycine, serotonin, dopamine, neurotransmitter), the present study examined the potential miRNA modulation of all predicted target mRNAs in the data set as well as the potential enrichment of modulated miRNAs and mRNAs within curated, canonical pathways using the QIAGEN Ingenuity^®^ Pathway Analysis (IPA) software.

All of the raw and normalized microarray and RNA-Seq expression data generated in this study have been deposited in the NCBI Gene Expression Omnibus (GEO accession # GSE60901, which includes microarray subseries GSE60819 and RNA-Seq subseries GSE60900).

### Molecular substrate analysis

The major finding from our previous behavioral study was that social motivation was significantly decreased in male and female rats prenatally exposed to ethanol at G12, and that this was reversed by social enrichment. The focus of the present study was to identify the molecular substrates underlying the social motivation deficit and its reversal. To accomplish this, our primary analysis utilized a 3 way ANOVA (2 genders × 2 prenatal diets × 2 postnatal treatments) for each brain region to identify miRNAs with highly consistent changes (1-tailed *p* < 0.1 for both RNA-Seq and microarray analyses) due to prenatal ethanol exposure and social enrichment. These ANOVAs were followed by Fisher’s *post hoc* testing to compare specific groups within each brain region. Notably, after identifying the miRNAs with the most robust main effects within each brain region, we also performed exploratory four-way ANOVAs, using the previous three factors plus brain region, and examined the top miRNAs for any evidence of significant interactions (e.g., brain region × diet, brain region × gender, brain region × social enrichment, and all other combinations of interactions). The results of this exploratory analysis are provided as Supplementary Material.

The top findings from the analysis of individual miRNAs were displayed in table format (Tables 1 and 2). We include in these results, individual miRNAs that were significantly changed (*p* < 0.05) according to the RNA-Seq analyses, but were not probed by the Affymetrix GeneChip miRNA 2.0 array.

**Table 1:** **Nominally significant miRNAs in amygdala**.

miRNA	miRBase 21 accession	RNA-Seq		Microarray	
		Fold change	*p*-Value	Fold change	*p*-Value
**ETHANOL EFFECT IN NON-ENRICHED RATS [ETHANOL_NON-SOCIAL_ VS CONTROL_NON-SOCIAL_]**					
miR-1843a-3p	MIMAT0024848	−1.50	0.097	−1.62	0.045
miR-221-5p	MIMAT0017163	−1.38	0.031	−1.15	0.004
miR-29c-3p	MIMAT0000803	−1.18	0.038	−1.12	0.068
miR-384-5p	MIMAT0005309	−1.12	0.063	−1.21	0.002
miR-412-3p	MIMAT0003124	−2.57	0.025	−1.20	0.032
mir-129-1	MI0000902	−1.31	0.079	−1.27	0.089
mir-138-2	MI0000911	−1.20	0.065	−1.28	0.014
mir-155	MI0025509	1.34	0.057	1.24	0.026
mir-322-2	MI0031763	−1.23	0.064	−1.36	0.017
mir-34c	MI0000876	2.83	0.072	1.52	0.013
mir-496	MI0012622	−1.54	0.010	−1.18	0.031
mir-9a-2	MI0000840	−1.17	0.062	−1.26	0.045
miR-148a-5p	MIMAT0035724	−1.19	0.011		
miR-15b-3p	MIMAT0017093	4.50	0.024		
miR-221-3p	MIMAT0000890	−1.27	0.010		
miR-222-3p	MIMAT0000891	−1.23	0.030		
miR-299a-5p	MIMAT0000901	−1.55	0.049		
miR-301b-3p	MIMAT0005304	2.85	0.017		
miR-448-3p	MIMAT0001534	4.07	0.040		
miR-449a-5p	MIMAT0001543	3.84	0.044		
miR-495	MIMAT0005320	−1.46	0.035		
miR-6329	MIMAT0025068	−2.38	0.003		
miR-667-3p	MIMAT0012852	−1.30	0.017		
mir-204	MI0000946	3.97	0.042		
mir-299a	MI0000970	−1.46	0.007		
mir-3084a	MI0030358	3.69	0.034		
mir-3556b-2	MI0031769	−1.18	0.006		
mir-448	MI0001639	3.59	0.050		
mir-6329	MI0021853	−1.45	0.045		
**ETHANOL EFFECT IN SOCIALLY ENRICHED RATS [ETHANOL_SOCIAL_ VS CONTROL_SOCIAL_]**					
mir-218a-2	MI0000957	1.33	0.054	1.22	0.029
let-7c-2	MI0000831	1.38	0.018		
let-7c-5p	MIMAT0000776	1.39	0.005		
miR-148a-5p	MIMAT0035724	−1.17	0.022		
miR-195-5p	MIMAT0000870	1.25	0.039		
miR-6319	MIMAT0025056	−11.33	0.002		
miR-6324	MIMAT0025063	−25.25	0.005		
miR-872-3p	MIMAT0005283	−1.39	0.002		
mir-195	MI0000939	1.26	0.044		
mir-6324	MI0021848	−4.38	0.008		
mir-708	MI0006160	1.25	0.030		
**SOCIAL ENRICHMENT EFFECT IN CONTROL RATS [SOCIAL_CONTROL_ VS NON-SOCIAL_CONTROL_]**					
miR-106b-5p	MIMAT0000825	−1.35	0.001	−1.39	0.008
miR-218a-5p	MIMAT0000888	−1.30	0.032	−2.15	0.010
miR-30c-5p	MIMAT0000804	−1.09	0.096	−1.39	0.002
miR-674-3p	MIMAT0005330	−1.29	0.024	−1.50	0.072
miR-96-5p	MIMAT0000818	−3.78	0.099	−1.32	0.001
miR-9a-3p	MIMAT0004708	−1.11	0.087	−1.51	1E-06
mir-218b	MI0015428	−1.31	0.036	−1.42	0.029
mir-503-2	MI0031773	−2.42	0.062	−1.46	0.001
mir-544	MI0012593	5.25	0.022	1.24	0.068
miR-106b-5p	MIMAT0000825	−1.35	0.001	−1.39	0.008
miR-218a-2-3p	MIMAT0004740	11.03	0.045		
miR-221-3p	MIMAT0000890	−1.20	0.034		
miR-299a-5p	MIMAT0000901	−1.57	0.044		
miR-3084d	MIMAT0035745	−1.84	0.041		
miR-503-3p	MIMAT0017224	−1.76	0.032		
miR-6319	MIMAT0025056	2.16	0.043		
miR-6329	MIMAT0025068	−1.58	0.042		
miR-667-3p	MIMAT0012852	−1.34	0.009		
miR-872-3p	MIMAT0005283	1.28	0.011		
mir-6319-1	MI0021841	4.26	0.028		
mir-708	MI0006160	−1.24	0.033		
**SOCIAL ENRICHMENT EFFECT IN ETHANOL RATS [SOCIAL_ETHANOL_ VS NON-SOCIAL_ETHANOL_]**					
miR-17-1-3p	MIMAT0004710	1.75	0.058	1.21	0.008
miR-204-5p	MIMAT0000877	−2.83	0.084	−3.52	0.057
miR-376b-5p	MIMAT0003195	1.36	0.015	2.29	9.7E-07
miR-378a-5p	MIMAT0003378	−1.98	0.014	−1.38	0.002
miR-384-5p	MIMAT0005309	1.12	0.060	1.12	0.038
miR-874-5p	MIMAT0017290	−1.91	0.047	−1.28	0.041
mir-19a	MI0000849	−1.88	0.079	−1.25	0.085
miR-142-3p	MIMAT0000848	−2.53	0.013		
miR-183-5p	MIMAT0000860	−358.64	0.049		
miR-199a-3p	MIMAT0004738	−1.51	0.027		
miR-222-3p	MIMAT0000891	1.21	0.049		
miR-301b-3p	MIMAT0005304	−2.21	0.041		
miR-3068-5p	MIMAT0024845	1.47	0.013		
miR-3557-3p	MIMAT0017820	−1.98	0.014		
miR-379-3p	MIMAT0004791	1.31	0.019		
miR-493-3p	MIMAT0003191	−2.80	0.050		
miR-6329	MIMAT0025068	1.84	0.049		
mir-296	MI0000967	1.45	0.034		
mir-299a	MI0000970	1.41	0.015		

*Comparisons and fold changes measured using RNA-Seq and, where available, validated by Microarray quantification. Table contains miRNAs with consistent changes at an uncorrected *p*-value cutoff of 0.1 or less for both RNA-Seq and Microarray data sets, or miRNAs that were not present on the array but were observed to change in the RNA-Seq data with an uncorrected *p-*value cutoff of 0.05 or less*.

**Table 2:** **Nominally significant miRNAs in ventral striatum**.

miRNA	miRBase 21 accession	RNA-Seq		Microarray	
		Fold change	*p*-Value	Fold change	*p*-Value
**ETHANOL EFFECT IN NON-ENRICHED RATS [ETHANOL_NON-SOCIAL_ VS CONTROL_NON-SOCIAL_]**					
let-7c-1	MI0000830	1.17	0.035	1.07	0.009
let-7c-2-3p	MIMAT0017088	1.91	0.041	1.17	0.084
mir-542-1	MI0003528	1.36	0.090	1.21	0.003
miR-1247-5p	MIMAT0035721	−1.79	0.047		
miR-133b-3p	MIMAT0003126	11.64	0.016		
miR-345-3p	MIMAT0004655	1.68	0.012		
miR-489-5p	MIMAT0017196	−2.00	0.032		
miR-493-3p	MIMAT0003191	−2.12	0.035		
miR-540-5p	MIMAT0017211	−1.31	0.030		
miR-6314	MIMAT0025047	2.35	0.047		
mir-122	MI0000891	−5.54	0.004		
mir-1306	MI0021537	−1.48	0.046		
mir-3591	MI0015471	−5.54	0.004		
mir-6314	MI0021832	2.36	0.044		
**ETHANOL EFFECT IN SOCIALLY ENRICHED RATS [ETHANOL_SOCIAL_ VS CONTROL_SOCIAL_]**					
miR-200b-3p	MIMAT0000875	4.55	0.065	3.94	0.053
miR-26b-3p	MIMAT0004714	1.55	0.041	1.08	0.012
mir-542-2	MI0031781	−1.34	0.037	−1.22	0.052
miR-133b-3p	MIMAT0003126	3.18	0.011		
miR-200a-3p	MIMAT0000874	6.48	0.044		
miR-344g	MIMAT0025052	−3.55	0.044		
miR-3553	MIMAT0017814	5.51	0.023		
miR-493-3p	MIMAT0003191	−2.03	0.026		
miR-532-3p	MIMAT0005323	−1.61	0.041		
miR-540-5p	MIMAT0017211	−1.27	0.023		
miR-582-5p	MIMAT0012833	−1.41	0.045		
mir-183	MI0000928	5.47	0.022		
mir-200a	MI0000943	6.74	0.048		
mir-3548	MI0015404	6.74	0.048		
mir-3553	MI0015410	5.47	0.022		
mir-3577	MI0015449	4.48	0.007		
**SOCIAL ENRICHMENT EFFECT IN CONTROL RATS [SOCIAL_CONTROL_ VS NON-SOCIAL_CONTROL_]**					
miR-155-5p	MIMAT0030409	1.61	0.098	1.47	2E-04
miR-24-2-5p	MIMAT0005441	1.14	0.085	1.28	0.031
miR-27a-5p	MIMAT0004715	3.55	0.038	1.31	2E-04
miR-299a-3p	MIMAT0017167	−1.54	0.018	−1.86	0.032
miR-434-5p	MIMAT0017307	1.11	0.030	1.45	1E-04
miR-487b-3p	MIMAT0003200	−1.15	0.037	−1.46	0.003
mir-1247	MI0030348	−1.81	0.041	−1.13	0.071
mir-31b	MI0015412	1.27	0.079	1.16	0.042
mir-487b	MI0003547	−1.14	0.033	−1.22	0.042
mir-653	MI0012600	−1.78	0.058	−1.41	0.011
mir-673	MI0006158	1.19	0.068	1.18	0.075
miR-1247-5p	MIMAT0035721	−1.80	0.045		
miR-145-5p	MIMAT0000851	1.49	0.017		
miR-224-5p	MIMAT0003119	11.41	0.019		
miR-3592	MIMAT0017895	1.63	0.005		
miR-3594-3p	MIMAT0017899	3.43	0.035		
miR-382-3p	MIMAT0003202	1.63	0.005		
miR-653-5p	MIMAT0012838	−1.84	0.038		
miR-98-5p	MIMAT0000819	−1.10	0.047		
mir-3577	MI0015449	−4.40	0.008		
**SOCIAL ENRICHMENT EFFECT IN ETHANOL RATS [SOCIAL_ETHANOL_ VS NON-SOCIAL_ETHANOL_]**					
miR-141-3p	MIMAT0000846	3.60	0.010	1.46	0.065
miR-182	MIMAT0005300	3.51	0.001	1.27	0.050
miR-200b-3p	MIMAT0000875	5.09	0.020	5.45	0.058
miR-320-3p	MIMAT0000903	−1.20	0.055	−1.27	0.093
miR-323-5p	MIMAT0004637	1.23	0.023	1.17	0.071
miR-874-5p	MIMAT0017290	−1.88	0.001	−1.60	0.099
mir-148a	MI0030350	−1.20	0.090	−1.21	0.076
miR-188-5p	MIMAT0005301	−4.04	0.019		
miR-200a-3p	MIMAT0000874	7.03	0.041		
miR-344g	MIMAT0025052	−3.64	0.038		
miR-3553	MIMAT0017814	4.55	0.029		
miR-381-5p	MIMAT0017220	3.47	0.047		
miR-532-3p	MIMAT0005323	−1.64	0.033		
miR-6318	MIMAT0025055	2.13	0.008		
mir-183	MI0000928	4.70	0.026		
mir-200a	MI0000943	6.81	0.048		
mir-3084a	MI0030358	2.82	0.032		
mir-344g	MI0021837	−1.88	0.012		
mir-3548	MI0015404	6.81	0.048		
mir-3553	MI0015410	4.70	0.026		

*Comparisons and fold changes measured using RNA-Seq and, where available, Microarray quantification. Conventions same as Table 1*.

A combined analysis of miRNA data and mRNA data was then performed. The mRNA targets of the most robustly affected miRNAs were mapped using the miRNA target filter workflow of QIAGEN Ingenuity^®^ Pathway Analysis (IPA) software. We also examined the entire mRNA dataset for specific canonical neuronal pathway effects using IPA software, using a threshold of *p* < 0.1 from the ANOVA *post hoc* testing.

We note that 0.1 was chosen as the *de facto* threshold for significance throughout most of our analyses because of the combined use of multiple platforms for cross-validation or the combined use of multiple genes within networks as well as miRNAs and their target mRNAs.

## Results

### microRNAs are altered by prenatal ethanol exposure and social enrichment

We performed a global screen of all known, curated miRNA molecules. To ensure full coverage, a conservative cross-platform approach employing both miRNA microarrays and RNA-Seq was used for identification of potential miRNA of interest. Quantification of miRNAs was based primarily on small RNA-Sequencing, which has increasingly emerged as the gold standard of miRNA quantification technologies, owing to its greater sensitivity and dynamic range compared to other techniques. Orthogonal validation was performed using Affymetrix miRNA GeneChips. The application of these two complementary technologies improved our capacity to discover relevant miRNAs that may have been overlooked had a single quantification method been employed. On the other hand, because miRNA microarrays are limited to the interrogated content of the arrays at the time of manufacture, we also included in our analyses those miRNAs that were found only by small RNA-Seq.

The Affymetrix GeneChip miRNA 2.0 array that we used included probes for 780 *Rattus norvegicus* precursor and mature miRNAs (representing approximately half that number of unique miRNAs). The RNA-Seq analysis that we employed identified 1063 precursor and mature miRNAs listed in the miRBase 21 annotation (18). A total of 601 miRNAs could be cross-referenced based on exact sequence conservation of the array probe and RNA-Seq annotation. In the space that follows, we describe first the changes due to fetal ethanol or postnatal social enrichment in these miRNAs, as seen in the amygdala and/or ventral striatum of both genders of rats.

#### Ethanol effects

In the amygdala, out of the 601 total miRNAs we identified a total of 291 miRNAs with consistent changes (in the same direction) due to ethanol in non-enriched animals representing 48% directional concordance. Of these, 12 miRNAs were changed in both platforms (at the *p* < 0.1 level) (Table 1, upper). An additional 17 miRNAs only found by RNA-Seq were also observed to change (at *p* < 0.05 level) due to ethanol effects in non-enriched rats (Table 1, upper). In rats subjected to social enrichment, we observed a total of 275 (46%) directionally concordant changes, with 1 miRNA changed (*p* < 0.1) in both platforms and 10 additional miRNAs significantly changed (*p* < 0.05) that were only found by RNA-Seq (Table 1, upper).

In the ventral striatum, 281 of the miRNAs (47%) showed concordant directional changes due to ethanol in non-enriched animals, with 3 changed (at the *p* < 0.1 level) in both platforms and 11 additional miRNAs significantly changed that were only found by RNA-Seq (Table 2, upper). In rats subjected to social enrichment, a total of 284 (47%) miRNAs showed directionally concordant changes, with 3 changed (*p* < 0.1) in both platforms, and 13 additional miRNAs significantly changed (*p* < 0.05) that were only found by RNA-Seq (Table 2, upper).

#### Social enrichment effects

For social enrichment effects in the amygdala of control rats, 251 (42%) miRNAs showed directional concordance with 9 miRNAs changed in both platforms at the *p* < 0.1 level and 11 additional miRNAs found by RNA-Seq (Table 1, lower). In corresponding ethanol-exposed rats, 286 (48%) showed directional concordance with 7 miRNAs changed in both platforms at the *p* < 0.1 level and 12 additional miRNAs found by RNA-Seq (Table 1, lower).

In the ventral striatum of control rats, 267 (44%) showed concordant directional changes due to enrichment, with 11 changed (at the *p* < 0.1 level) in both platforms and 9 additional miRNAs changed (at the *p* < 0.05 level) by RNA-Seq (Table 2, lower). In corresponding ethanol-exposed rats, 273 (45%) showed directional concordance with 7 miRNAs changed in both platforms at the *p* < 0.1 level and 13 additional miRNAs found by RNA-Seq (Table 2, lower).

We note that most of the 53 concordant miRNA differences found by both miRNA and RNA-Seq were similar in magnitude. However, the magnitude of the difference found by RNA-Seq exceeded the difference found by microarray by at least 50% for 10 miRNAs, while the difference by array was 50% greater than RNA-Seq for only 2 miRNAs. These observations lend additional support for the growing awareness that RNA-Seq appears to have greater dynamic range than microarray-based expression profiling.

**Figure 2:**
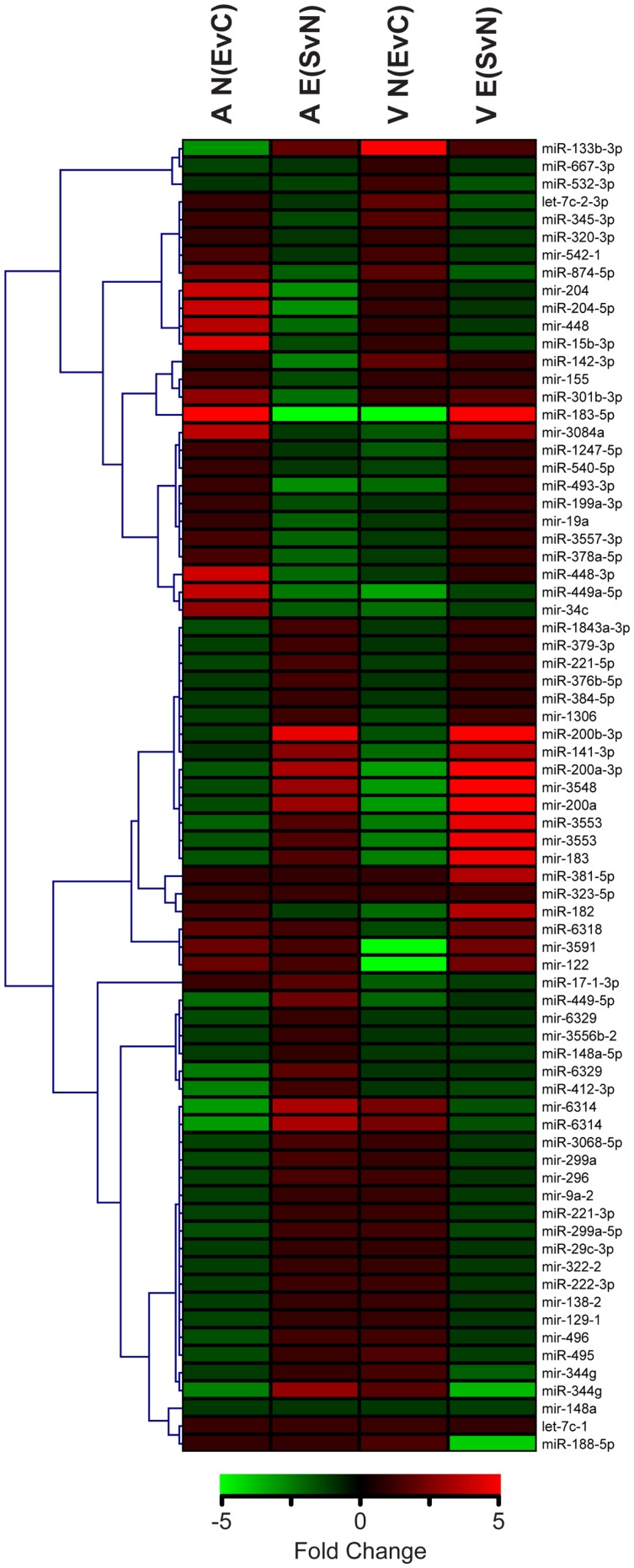
**Clustering of microRNAs with ethanol and/or social enrichment effects**. Hierarchical clustering analysis of fold change values in miRNAs with significant ethanol effects in non-enriched rats [N(EvC)] and social enrichment effect in ethanol rats [E(SvN)] for both brain regions [A or V].

From this point forward, we specifically chose to further examine the ethanol effect in non-enriched rats [N (EvC)] and the social enrichment effect in ethanol-exposed rats [E (SvN)], as these groups exhibited a striking reversal in social motivation (5). Hierarchical cluster analysis showed distinct expression patterns in groups of miRNAs, including some with directional reversals resulting from social enrichment (Figure 2). Except for one (miR-381-5p), none of the miRNAs in this subset showed any main effects of gender (Datasheet S1 in Supplementary Material). Thus, they do not appear to have gender-specific gene effects.

This comparative analysis also revealed several notable individual miRNAs (Table 3). First, miR-874-5p was decreased in both the amygdala and ventral striatum. On the other hand, mir-183 was affected by social enrichment in both brain regions, with its mature miR-183-5p showing a striking 300-fold decrease in the amygdala and 5-fold increase in the ventral striatum. Thus, brain region clearly influenced the miRNA results.

**Table 3:** **Notable miRNA comparisons**.

miRNA	miRBase 21 accession	Comparison	RNA-Seq		Microarray	
			Fold change	*p*-Value	Fold change	*p*-Value
**DECREASED BY ETHANOL IN BOTH REGIONS**						
miR-874-5p	MIMAT0017290	A E(SvN)	−1.91	0.047	−1.28	0.041
miR-874-5p	MIMAT0017290	V E(SvN)	−1.88	0.099	−1.60	0.001
**AFFECTED BY SOCIAL ENRICHMENT IN BOTH REGIONS**						
miR-183-5p	MIMAT0000860	A E(SvN)	−358.64	0.049		
mir-183	MI0000928	V E(SvN)	4.70	0.026		
**MICRORNA-122 FAMILY AFFECTED BY ETHANOL IN VENTRAL STRIATUM**						
mir-3591	MI0015471	V N(EvC)	−5.54	0.004		
mir-122	MI0000891	V N(EvC)	−5.54	0.004		
**MICRORNA-8 FAMILY AFFECTED BY SOCIAL ENRICHMENT IN VENTRAL STRIATUM**						
mir-200a	MI0000943	V E(SvN)	6.81	0.048		
miR-200a-3p	MIMAT0000874	V E(SvN)	7.03	0.041		
miR-200b-3p	MIMAT0000875	V E(SvN)	5.09	0.058	5.45	0.020
miR-141-3p	MIMAT0000846	V E(SvN)	3.60	0.065	1.46	0.010
mir-3548	MI0015404	V E(SvN)	6.81	0.048		
**REVERSED BY SOCIAL ENRICHMENT IN AMYGDALA**						
mir-204	MI0000946	A N(EvC)	3.97	0.042		
miR-204-5p	MIMAT0000877	A E(SvN)	−2.83	0.084	−3.52	0.057
miR-384-5p	MIMAT0005309	A N(EvC)	−1.12	0.063	−1.21	0.002
miR-384-5p	MIMAT0005309	A E(SvN)	1.12	0.060	1.12	0.038
miR-222-3p	MIMAT0000891	A N(EvC)	−1.23	0.030		
miR-222-3p	MIMAT0000891	A E(SvN)	1.21	0.049		
mir-299a	MI0000970	A N(EvC)	−1.46	0.007		
mir-299a	MI0000970	A E(SvN)	1.41	0.015		
miR-301b-3p	MIMAT0005304	A N(EvC)	2.85	0.017		
miR-301b-3p	MIMAT0005304	A E(SvN)	−2.21	0.041		
miR-6329	MIMAT0025068	A N(EvC)	−2.38	0.003		
miR-6329	MIMAT0025068	A E(SvN)	1.84	0.049		

*Conventions same as Table 1. A N(EvC)-ethanol effect in non-enriched rats’ amygdala; A E(SvN)-social enrichment effect in ethanol rats’ amygdala; V N(EvC)-ethanol effect in non-enriched rats’ ventral striatum; V E(SvN)-social enrichment effect in ethanol rats’ ventral striatum*.

In addition to single miRNAs, we also examined whether our miRNAs of interest belonged to the same miRBase families, which are clusters of highly homologous sequences. Interestingly, the miRNA families that had common members, including mir-122 (miRBase family accession # MIPF0000095) and mir-8 (miRBase family accession # MIPF0000019) had very similar fold changes and *p*-values within the same comparisons. We note that the identical results between members of the same miRNA family likely reflect the fact that our RNA-Seq analysis could not distinguish the two isoforms using the standard read count quantification algorithm that we employed. Additional experiments on precursor forms of these miRNAs would be needed to elucidate the effects of individual miRNAs within such families. This would be particularly interesting for the mir-8 family, whose members have been implicated in synaptic development (19).

Most importantly, we also noted several miRNAs that appeared to significantly reverse their expression levels in the amygdala after social enrichment in ethanol-exposed animals. These include precursor miRNAs mir-204 and mir-299a as well as mature miRNAs miR384-5p, miR-222-3p, and miR-301b-3p. Because these molecular changes parallel the behavioral changes, it is possible that they may be more directly related to the primary mechanisms underlying each phenotypic effect.

### Differential modulation of targeted messenger RNAs converge on cell signaling and morphology

In order to elucidate the large-scale functional changes being affected by ethanol and social enrichment, we performed functional network enrichment analysis using QIAGEN Ingenuity^®^ IPA software. The IPA core analysis workflow determines key gene regulatory networks that are overrepresented in any given set of molecules. Additionally, the activation or inhibition of upstream and downstream molecules can be predicted based on existing data and overlaid on any network to show how the overall network is affected.

**Figure 3:**
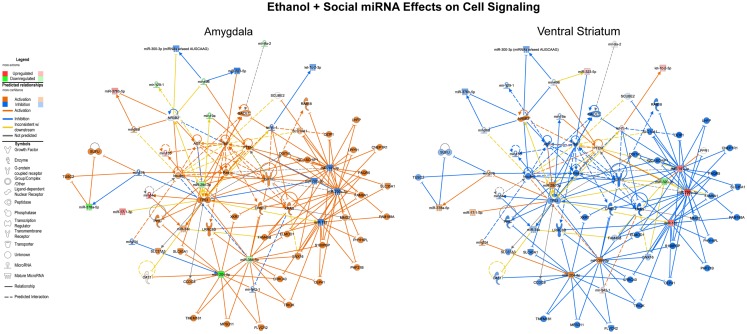
**Ethanol and social enrichment effects on microRNAs**. The results of a gene network level analysis of all of the miRNAs with changes in one or more of the contrasts listed in Tables 1 and 2 are shown. Genes and miRNAs with increased or predicted increased expression are shown in red and orange, respectively, while genes and miRNAs with decreased or predicted decreased expression are shown in green and blue, respectively. Genes and miRNAs with conflicting predicted vs observed data are connected by yellow lines, and genes and miRNAs with absent data or unpredicted relationships are unfilled and connected by gray lines. Note that in general, the network is activated in the amygdala and inhibited in the ventral striatum.

We first performed this analysis using only the specific miRNAs validated using both RNA-Seq and array that were altered by ethanol in non-social animals and by social enrichment in ethanol-exposed animals (Tables 1 and 2). The combined networks obtained by a core analysis of these data were merged and examined using data from the amygdala and ventral striatum. Overall, the functions represented by the resulting merged network included several cell signaling molecules. Hereafter, we refer to this merged network as a Cell Signaling network (Figure 3). Key hub molecules in this network of miRNAs and target mRNAs include p53, IGF1R, TNF, and several others. Most interestingly, the molecule activity predictor tool in IPA generally suggested a large-scale activation (orange colors) of this network in the amygdala and inhibition (blue colors) in the ventral striatum.

The miRNA data were also integrated with mRNA data (*p* < 0.1) derived from the same tissues (5) using the IPA miRNA target filter workflow. Relationships included experimentally determined data from the Ingenuity^®^ curated database and highly predicted targets from the target prediction databases in TargetScan (20), miRecords (21), and TarBase (22). We note that the miRNA-target predictions are based on sequence complementarity between the miRNA seed sequence and the target mRNA, and thus may be applicable to several miRNAs with the same seed sequence. In this report, we include findings from mRNA targets that have opposing expression level changes to their predicted miRNA regulators (Datasheet S2 in Supplementary Material). Because of their large-scale nature, these results were exclusively examined at the network level.

#### Ethanol effects reversed by social enrichment

**Figure 4:**
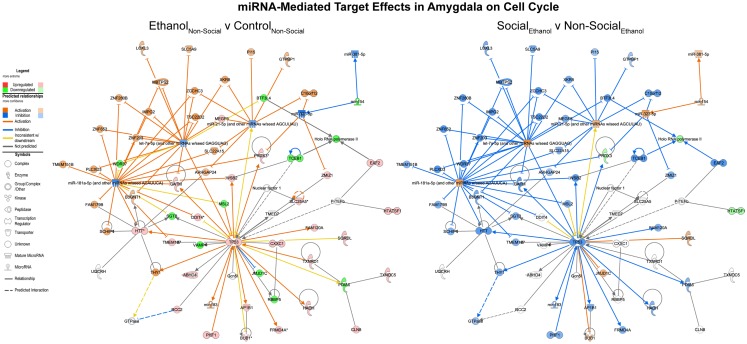
**MicroRNA target effects in the amygdala on cell cycle signaling**. Conventions same as Figure 3. Note that nearly all of the genes show a distinct reversal in their expression difference following social enrichment.

Focusing on the combined miRNA-mRNA target networks in the amygdala, the first major network identified was one involved in cell cycle processes (Figure 4, left). This network generally contained RNAs, which were inhibited following prenatal ethanol (e.g., VAMP4, mir-154). Other genes within the network showed increased expression, including p53. Remarkably, almost across the board, the pattern of changes in these genes due to prenatal ethanol was completely opposite the changes seen following social enrichment (Figure 4, right).

**Figure 5:**
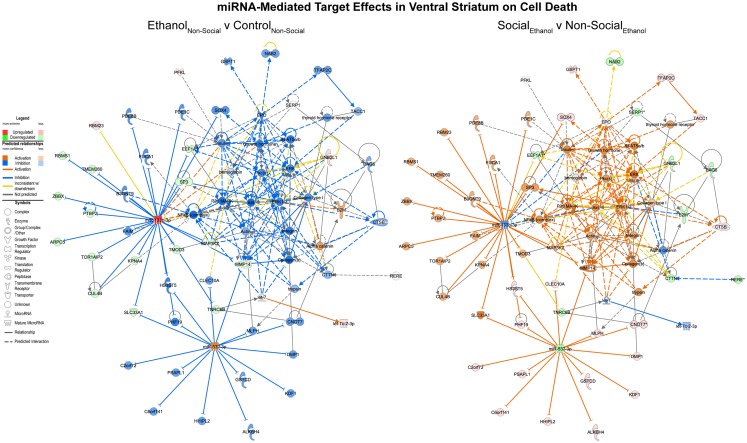
**MicroRNA target effects in the ventral striatum on cell death signaling**. Conventions same as Figure 3. Note that nearly all of the genes show a distinct reversal in their expression difference following social enrichment.

On the other hand, corresponding ethanol effects in the ventral striatum revolved around cell death processes with inhibition of RNAs including MAP3K2 and upregulation of RNAs like let-7 (Figure 5, left). These are predicted to inhibit cell death genes like AKT and ERK1/2. Again, the network appears activated as a result of social enrichment, resulting from downregulation of molecules such as miR-532-3p (Figure 5, right).

### Canonical gene expression pathways in neurons are altered by ethanol and reversed by social enrichment

In addition to the networks identified above, we also chose to extend our previously reported characterization of 660 brain-related mRNAs of interest (5) by examining the evidence for network level changes in four curated canonical IPA pathways: p53 signaling, GABA receptor signaling, glutamate receptor signaling, and CREB signaling in neurons.

**Figure 6:**
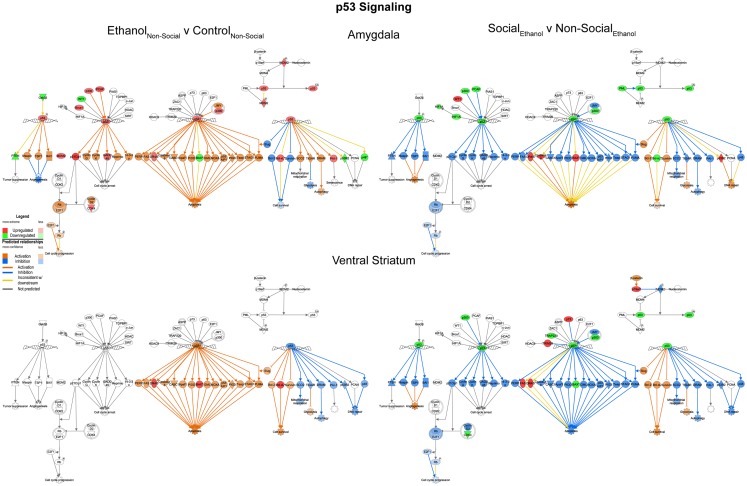
**Ethanol and social enrichment effects on nuclear p53 signaling**. Conventions same as Figure 3. Note that the changes in p53 signaling were highly similar in both brain areas following prenatal ethanol exposure (left), but showed differential responsiveness in the two brain areas following social enrichment (right).

We examined the p53 signaling pathway because of differential responses between prenatal ethanol exposure and social enrichment (Figure 6). This network generally showed robust increases in expression following prenatal ethanol exposures in both the amygdala and ventral striatum. Following social enrichment, most of the genes in this network showed decreased expression in the ventral striatum, with a smaller subset showing decreased expression in the amygdala. Thus, the ventral striatum appeared to be more differentially responsive to the social enrichment effects on p53 signaling. Notably, we and others have consistently observed highly consistent changes in p53/apoptosis signaling networks following ethanol exposure (23, 24). However, this is the first report we are aware of to report changes in p53 signaling genes following social enrichment.

We also examined evidence for changes in GABA, glutamate, and CREB signaling. Our rationale for doing so was based on the fact that ethanol acts as a GABA agonist and NMDA antagonist, and has well-characterized effects on CREB signaling within specific brain circuits involved in addiction, including the ventral tegmental area, striatum, and cortex [reviewed in Ref. (25)]. Furthermore, GABA and glutamate neurotransmitter systems have been shown to be differentially expressed in alcohol-preferring vs non-preferring rats (26) and alcohol’s effects on the central amygdala are known to affect glutamatergic and GABAergic transmission as a result of acute exposure [reviewed in Ref. (27)].

#### GABA receptor signaling

**Figure 7:**
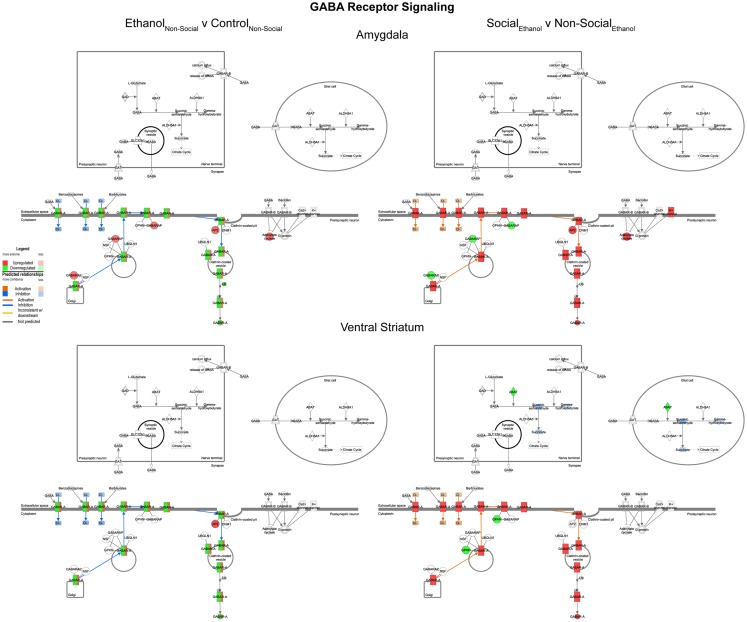
**Ethanol and social enrichment effects on GABA receptor signaling**. Conventions same as Figure 3. Note that prenatal ethanol exposure generally inhibits GABA signaling and is reversed following social enrichment. In some cases, due to the presence of multiple isoforms of a gene with the same name, multiple colors are contained within a symbol.

In general, we observed trends for decreased expression of multiple GABA related transcripts following prenatal ethanol exposure in both the amygdala and ventral striatum (Figure 7, left). These trends were consistently reversed after social enrichment (Figure 7, right). These observations suggest a plastic mechanism is in place within the basal forebrain. Markers for GABAergic neurons have been found in the amygdala as early as G20 in rats (28) suggesting that this system could be responsive throughout much of the animal’s lifetime.

#### Glutamate receptor signaling

**Figure 8:**
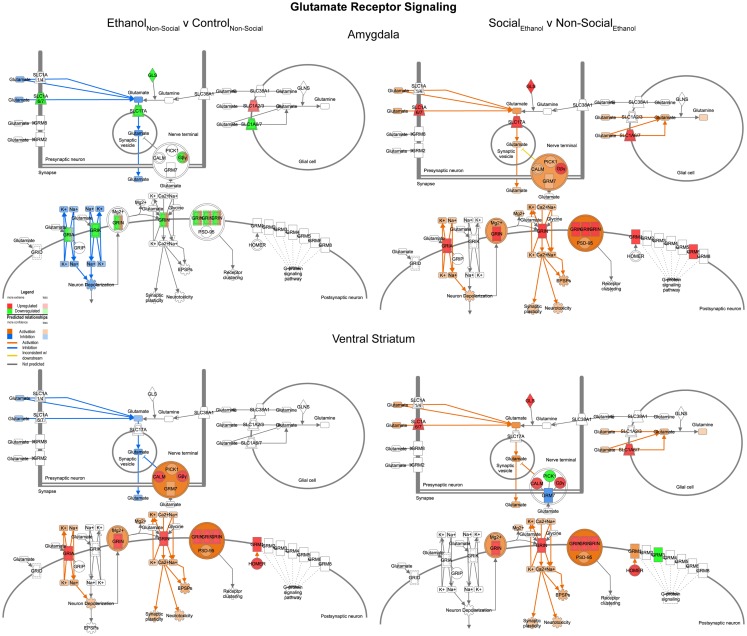
**Ethanol and social enrichment effects on glutamate receptor signaling**. Conventions same as Figure 3. Note that this network tended to show differential changes due to prenatal ethanol exposure in the two brain areas (left), but somewhat more consistent changes following social enrichment (right).

In contrast to the changes seen for GABA networks, we found evidence of region-specific changes in glutamate related genes. Specifically, prenatal ethanol exposure was associated with generally decreased expression in the amygdala and generally increased expression in the ventral striatum (Figure 8, left). However, following social enrichment, both of the brain areas tended to show large-scale increases in expression (Figure 8, right). These differences suggest that changes in glutamate signaling in the amygdala may be more directly linked to the social behavioral deficits we have observed, while changes in the ventral striatum may be more reflective of exposure to a drug of abuse. Furthermore, the ethanol findings are also consistent with observations on the acute effects of ethanol on glutamate receptor function [reviewed in Ref. (29)]. Our findings also suggest that glutamate receptor-mediated synaptic plasticity is altered, particularly in the amygdala, consistent with was has been reported for ethanol effects in the hippocampus (30).

#### CREB signaling in neurons

**Figure 9:**
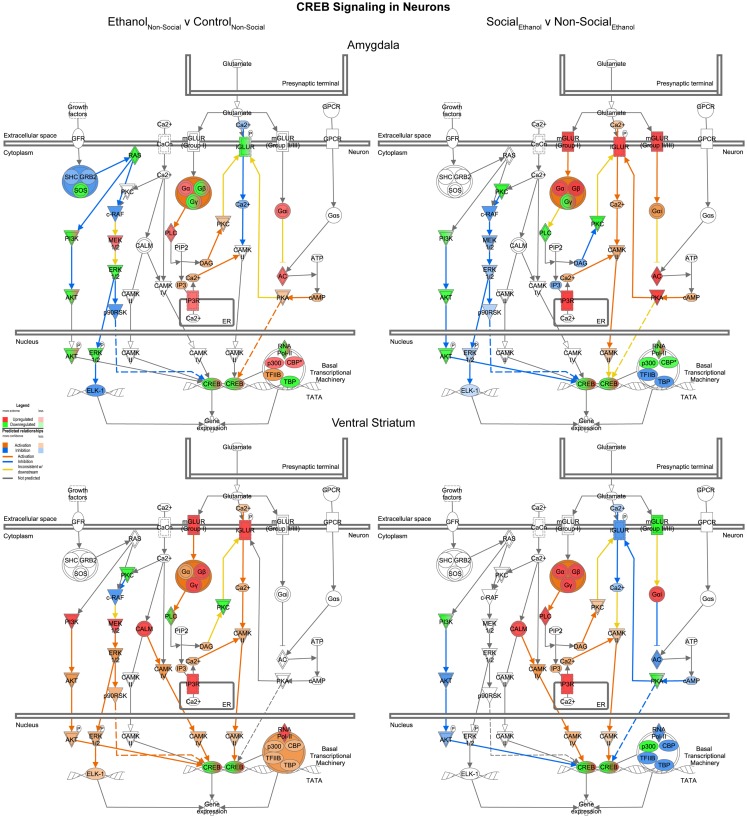
**Ethanol and social enrichment effects on CREB signaling in neurons**. Conventions same as Figure 3. Note that the changes produced following prenatal ethanol exposure or social enrichment showed somewhat complex patterns, with some evidence of both region-specific and treatment-specific effects.

Reinforcing the changes just described for glutamate signaling, the last network we examined was one involved in CREB signaling in neurons. In this case, we found it much more difficult to generalize about one specific direction of change within this highly integrated cellular network across the two brain areas. Indeed, following prenatal ethanol exposure, more than 10 genes showed changes in distinctly opposite directions in the amygdala and ventral striatum (e.g., IGLUR, Gβ, Gγ, PLC, PKC, AKT, ERK, p90RSK, p300, CBP, TFIIB, TBP) (Figure 9, left). Following social enrichment, however, there was somewhat greater agreement between the two brain areas in the directionality (or predicted directionality) of the changes (Figure 9, right). These findings underscore the importance of examining entire transcriptional networks before reaching conclusions regarding the potential effect that a manipulation or treatment may have in a specific brain region. Moreover, the results highlight the utility of examining multiple brain regions.

## Discussion

This study sought to evaluate molecular mechanisms at the miRNA, mRNA, and gene regulatory network levels that underlie the effective reversal of a social motivation deficit seen following prenatal ethanol exposure (5). We found several robustly affected miRNAs, target mRNAs, and functional pathways that could represent candidate control points for the behavioral deficits we previously observed.

Several recent miRNA studies have been performed on rodents or primary cell cultures exposed to ethanol either during gestational or postnatal time periods. We compared the most robustly changed miRNAs in our studies (Tables 1 and 2) to results from these other studies. Interestingly, 24 of the 48 miRNAs we observed with changes due to ethanol in either the amygdala or ventral striatum were also reported to change in other studies. Of those, 9 miRNAs (let-7c-1, miR-221-3p, miR-221-5p, miR-222-3p, mir-322-2, mir-34c, miR-384-5p, mir-496, and mir-542-1) reported consistent directional changes as our data (15, 31–33). This is despite the use of different exposure paradigms, brain areas, and cell types, as well as different species. These miRNAs may thus represent highly robust and persistent indices of ethanol exposure.

Most importantly, two of these microRNAs (miR-222-3p and miR-384-5p) were also found to be reversed in the amygdala after social enrichment (Table 3). By targeting the PTEN gene, mir-222 has been shown to promote neurite outgrowth (34). Additionally, mir-384 has been shown to be an indicator of neurotoxicity (35) and was found to be differentially expressed in dopaminergic neurons following cocaine addiction (36).

On the other hand, 7 miRNAs (mir-138-2, miR-148a-5p, mir-299a, miR-299a-5p, miR-493-3p, miR-540-5p, and miR-667-3p) have reported significant changes in miRNAs that only show directional changes opposite to what we observed (31, 32, 37, 38). Finally, there is mixed support for 7 of the miRNAs we reported (mir-129-1, miR-15b-3p, mir-204, miR-29c-3p, miR-301b-3p, miR-495, and mir-9a-2), with some studies showing changes consistent with our data, and other studies showing changes opposite those of our study (15, 31–33, 38–47). It is important to note that mir-9 has well-established roles in neurogenesis [reviewed in Ref. (48)]. Possible explanations for the disparities in results are likely found in the parameters of those studies. Regardless, these latter two sets of miRNAs may represent less-reliable or less persistent biomarkers of ethanol exposure.

Rather than focusing on individual genes, our large-scale analyses of ethanol-induced changes in miRNA and mRNA expression focus on whether effects on functionally related pathways are consistently seen across studies. From this perspective, our results add substantial support to the concept of a systems-level disruption of major gene regulatory pathways by a common environmental insult. We show in our study that prenatal alcohol exposure imposes a long-lasting effect on neuronal and, ultimately, behavioral function in adolescents. Notably, our results also extend our previous molecular analyses by incorporating the vast post-transcriptional regulatory aspects embodied by microRNAs. By evaluating concurrent changes in miRNA and mRNA levels, this work shines light on an additional layer of complexity to the gene expression changes occurring in the amygdala and ventral striatum. This is critical because miRNAs are thought to respond greatly to environmental stressors and are thought to mediate global gene expression changes [reviewed in Ref. (49, 50)].

Notably, the functions represented by the pathways represented in Figures 3–5 clearly implicate alterations in p53 signaling, cell cycle, and cell death pathways as a consequence of prenatal ethanol exposure. These pathways are of particular note because they suggest that abnormal cellular proliferation and/or DNA damage repair processes could be associated with early ethanol exposures. Indeed, we previously reported robust changes in genes involved in these processes in adult human alcohol abusing subjects (23). Although there is evidence that cortical heterotopias can occur as a consequence of early ethanol exposure, data from several human studies have failed to demonstrate any consistent elevation in the risk for childhood cancers, with some studies even reporting protective effects (51, 52).

To our knowledge, this is the first report of miRNA-directed gene expression changes brought about by environmental interventions in any FASD model. The potential reversal of abnormal changes in miRNA and mRNA expression by a relatively simple intervention (social enrichment) is consistent with data from other disorders, where specific changes in miRNA levels have been seen to result from an enriched environment, corresponding with slowing of the disease progression and improvement in hippocampal synaptic transmission using an Alzheimer’s disease model (53). Given the considerable data showing that environmental enrichment is likely one of the most effective means of improving outcomes in children with FASD and autism spectrum disorders, it is highly likely that such interventions exert at least some of their therapeutic effects through alterations in miRNA and mRNA levels in some of the same brain circuits we examined in this report.

It is beyond the scope of the present report to fully examine the evidence for regionally specific changes in expression. However, we note that both at the individual miRNA level and target mRNA level, many of the changes appeared to be region specific. This was even more evident for some of the comparisons made for specific functional pathways. Taken together, these patterns reinforce other recent findings, such as those by Tapocik et al. (54), who showed that mir-206 upregulation due to ethanol is regionally selective in the medial prefrontal cortex of a rat model of alcohol dependence and is not found in the amygdala or other regions of the brain. Clearly, much additional work will be needed to create comprehensive profiles for all of the brain-wide changes seen following prenatal ethanol exposure or social enrichment.

There are several limitations to note in the present study. First, it is important to acknowledge that the resulting gene expression effects we have observed in P42 rat brains reflect the cumulative effect of all life experiences to that age. That is, everything that the animal has experienced could alter miRNA and mRNA expression patterns; consequently, some of these alterations could interact with variables that were outside our ability to control. We tried to minimize the differences due to random noise that might exist between treatment groups. Nonetheless, in our study, pregnant animals ware received in the lab on G4, handled and injected on G12, potentially causing gestational stress. Exposure of the offspring to the anesthetic agents ketamine and xylazine immediately prior to decapitation may also have altered gene expression in some manner. It is possible that the treatment itself (ethanol exposure) interacted with the pre- or postnatal stressors, which include shipment, handling, and anesthetic administration. We note that the time course by which the anesthetics acted (ketamine/xylazine) is extremely brief (lasting only a few minutes prior to decapitation and dissection) and thus is not likely to create any large-scale biases in gene expression differences.

Another limitation in the present study is the lack of correction for multiple testing. We contend that our use of two independent quantification methodologies somewhat mitigates this concern. Furthermore, our focus on functional gene network analysis, rather than individual miRNAs and mRNAs *per se* also helps reduce concern about type 1 error. The seemingly low concurrency of RNA-Seq and array data may be the result of several factors, most notably the use of 2 different miRBase databases in our high-throughput quantification (array used miRBase 15, while sequencing uses miRBase 21). In addition, we used very stringent concurrency criteria because it was based on exact sequence homology between the array probe and the gene annotation against which the RNA-Seq data were quantified upon.

In conclusion, despite some limitations, our data strongly demonstrate that prenatal ethanol exposure has the capacity to impart long-lasting gene expression changes at both the miRNA and subsequent target mRNA level. Some of these changes clearly impact large-scale functional pathways in the brain that are involved in synaptic function and intracellular signaling, as well as cell cycle regulation, and brain development. Further studies are necessary to determine the extent to which changes in these pathways represent points of no return, or novel therapeutic opportunities for intervention.

## Conflict of Interest Statement

The authors declare that the research was conducted in the absence of any commercial or financial relationships that could be construed as a potential conflict of interest.

## Supplementary Material

The Supplementary Material for this article can be found online at http://www.frontiersin.org/Journal/10.3389/fped.2014.00103/abstract

Datasheet S1 ***p*-values for main and interaction effects in miRNAs of interest**. These include miRNAs with significant ethanol effects in non-enriched rats [N(EvC)] and social enrichment effect in ethanol rats [E(SvN)] from both brain regions.

Datasheet S2 **Messenger RNA targets from group comparisons**. Targets were filtered on opposing expression pairing between miRNAs and corresponding mRNA targets. Genes with functions related to neurological disease are highlighted in green. Microarray and RNA-Seq expression data have been deposited in the NCBI Gene Expression Omnibus (GEO accession # GSE60901, which includes microarray subseries GSE60819 and RNA-Seq subseries GSE60900).
